# Simultaneous Determination of Catalpol, Aucubin, and Geniposidic Acid in Different Developmental Stages of* Rehmannia glutinosa* Leaves by High Performance Liquid Chromatography

**DOI:** 10.1155/2016/4956589

**Published:** 2016-06-26

**Authors:** Yanjie Wang, Dengqun Liao, Minjian Qin, Xian'en Li

**Affiliations:** ^1^Institute of Medicinal Plant Development, Chinese Academy of Medical Sciences and Peking Union Medical College, Beijing 100193, China; ^2^Department of Resources Science of Traditional Chinese Medicines, China Pharmaceutical University, Nanjing 210009, China

## Abstract

Although* R. glutinosa* roots are currently the only organ source in clinics, its leaves are a potential supplement for the roots especially in extraction of some important bioactive compounds. Our early work found that the contents of catalpol and total iridoid glycosides varied among different developmental stages of* R. glutinosa* leaves. Aucubin and geniposidic acid, the abundant major bioactive compounds in* Eucommia ulmoides* and* Gardenia jasminoides*, respectively, were found present in* R. glutinosa* roots, however, and have not been analyzed in its leaves. In this paper, we aimed to determine contents of these three iridoid glycosides in different developmental stages of* R. glutinosa* leaves using the optimized HPLC-UV conditions. Our results showed that aucubin and GPA in* R. glutinosa* leaves were much lower than catalpol and showed the increasing trend with the leaf development, which was different from catalpol. This work provided the important information for future exploitation of* R. glutinosa* leaves as a potential supplement for its roots in extraction of some important bioactive compounds and studying the relationship of aucubin and catalpol metabolism.

## 1. Introduction

*Rehmannia glutinosa* Libosch (dihuang in Chinese) belongs to the family Scrophulariaceae. Its raw or processed roots are the only organ source for clinical uses of traditional Chinese medicine, and the leaves are not utilized at all. However,* R. glutinosa* leaves have the pharmacological effects and can nourish yin, tonify qi and kidney, and promote blood circulation [1]. In the folk, the fresh leaves are often applied externally to treat malignant sore and tinea manus and pedis [2]. As a perennial herb,* R. glutinosa* has to be annually cultivated and harvested since its fleshy roots easily decay during the winter dormancy or are consumed when new plantlets come out from them in the next season. Due to its severe continuous cropping obstacle, however,* R. glutinosa* plants could not grow well on the same land after the first cropping, decreasing the root yield greatly [3]. Thus, exploitation of* R. glutinosa* leaves not only provides another potential medicinal source for extraction of important bioactive compounds present in its roots but also complements the shortage of* R. glutinosa* roots due to the limited land and continuous cropping obstacle.

A lot of pharmacologically bioactive secondary metabolites [4, 5] including iridoid glycosides, phenylethanoid glycosides, and polysaccharides have been isolated in* R. glutinosa*, of which iridoid glycosides are thought to be main bioactive constituents. Till now, more than thirty iridoid glycosides have been separated and identified [4–6], including catalpol, aucubin, geniposidic acid (GPA), rehmanniosides A, B, C, and D, and rehmaglutosides A–K. The pharmacological effects of some of these iridoid glycosides have been extensively studied. For example, as the main active iridoid glycoside in* R. glutinosa*, catalpol was found to play the important roles in treatment of many diseases including kidney diseases [7], neurodegenerative diseases [8], and diabetes [9, 10]. It is revealed that aucubin had pharmacological effects such as antifungals [11], anti-inflammation and antioxidation [12], and hepatoprotection [13]. Similarly, GPA had therapeutic effects in anti-inflammation, liver disorders, and antinociception [14, 15]. Among these three compounds, only catalpol was assayed in detail to reveal its spatiotemporal expression profiling among different developmental stages of* R. glutinosa* leaves and between leaf and root [16, 17]. It is shown that catalpol content varied among different developmental stages of* R. glutinosa* leaves and within the whole growth stage. Aucubin and GPA were found to accumulate in roots of* R. glutinosa* [18, 19], and only the former was quantified and much lower than catalpol in the root [18, 20]. Therefore, in this paper, we aimed to determine the content of these three iridoid glycosides of two* R. glutinosa* cultivars in different developmental stages of leaves using HPLC-UV method and compared their changing trend.

## 2. Materials and Methods

### 2.1. Plant Material

Two primary cultivars of* R. glutinosa*, Wen 85-5 and Beijing no. 1, were grown in around mid-April in 2014 at the IMPLAD (Institute of Medicinal Plant Development), Beijing, China. The plant interval was 40 cm × 40 cm. Normal field management was applied during the growth period. On 11th September, 2014, the top eight positions of leaves from each plant were collected separately, washed to remove the surface soil, and then stored at −80°C till use.

### 2.2. Reagents and Standard Solution Preparation

HPLC grade acetonitrile (ACN) and formic acid were purchased from Thermo Fisher (USA). The standard catalpol (≥98%) was obtained from the National Institute for Food and Drug Control, China (http://www.nifdc.org.cn/). Standards of aucubin (GR-133-140104) and geniposidic acid (GR-133-140423) were purchased from Nanjing Guangrun Biotechnology Co. Ltd., China (http://www.grbiology.com/). The purified water from Wahaha was used throughout the experiment. Other reagents of analytical grade were from Beijing Chemical Industry Inc., China.

The concentrations of catalpol, aucubin, and geniposidic acid in the mixed working stock standard solution were, separately, 0.5025 mg, 0.0375 mg, and 0.0020 mg in 1 mL of purified water. To make calibration curves, six series concentrations, ranging from 0.01570~0.5025 mg/mL for CA, 0.001172~0.0375 mg/mL for AU, and 6.281*E* − 05~0.002010 mg/mL for GPA, were then separately prepared by diluting the stock working solution with twofold difference. Calibration curves were constructed by plotting the logarithm of the HPLC-UV peak areas versus the logarithm concentration of each standard.

### 2.3. Extraction and Assay of Three Iridoid Glycosides in* R. glutinosa* Leaves

The frozen samples were homogenized with liquid nitrogen and one gram of each sample powder was extracted twice with 25 mL of 30% methanol in an ultrasonic water bath at 25°C for 20 min. The extract was centrifuged at 12000 rpm for 5 min. The supernatants from two cycles of extraction were combined and evaporated to dryness in a rotary evaporator at 50°C under the reduced pressure, redissolved in 50 mL of pure water, and then filtered with a 0.22 *μ*m Millipore membrane filter prior to HPLC analysis.

For HPLC-UV quantification, ten microliters of filtered extracts and standards was run at 30°C on a waters 600E system equipped with a Phenomenex Kinetex C18 column (4.6 mm × 100 mm, 2.6 *μ*m), waters 2487 dual wavelength detector, and 2707 autosampler (USA). The isocratic mobile phase contained acetonitrile (5%) and 0.1% formic acid in water (95%) and was delivered at 0.4 mL/min. Catalpol and aucubin were monitored at *λ* 210 nm and geniposidic acid was monitored at *λ* 240 nm. The presence of the three iridoid glycosides in* R. glutinosa* leaves was determined by comparing both retention time and spectral data with those of their corresponding authentic standards. Concentrations of three iridoid glycosides in the samples were determined from the below linear standard calibration curves. Their content was shown in dry weight. Since catalpol and other iridoids are not heat-stable, the batch of leaf samples was used to calculate the dry weight of each stage leaf. Three replicates of each stage of leaf sample were measured.

## 3. Results and Discussion

### 3.1. Optimization of HPLC-UV Conditions

HPLC-UV method has been successfully applied to quantify catalpol (CA), aucubin (AU), and GPA in medicinal plants [16, 17, 20–22]. The HPLC conditions that researchers used varied in terms of the columns, mobile phase system, and detection wavelengths. Therefore, we optimized HPLC-UV conditions in terms of column type, UV wavelength, mobile phase composition, addition of formic acid in aqueous phase, and ratio of ACN and water in our early work (Table 1). Several UV wavelengths (e.g., 203 nm, 205 nm, 206 nm, and 210 nm) were used to detect the presence and content of catalpol and (or) aucubin. We found that there was no significant difference in detecting these two iridoid glycosides under 203~210 nm and thus chose 210 nm as the detection wavelength, which was used in* China pharmacopoeia* (2010). There was no much difference of the wavelengths (230~240 nm) in peak shape and separation of GPA except that less miscellaneous peaks and stable baselines were produced at *λ* 240 nm. Thus, GPA was detected at *λ* 240 nm in latter experiments. As Ji et al. [16] revealed, adding or not adding formic acid (FA) in the mobile phase did not obviously influence the peak shape and separation of catalpol and aucubin, except for GPA. While investigating the difference of different concentrations of ACN in the mobile phase system on the chromatogram, we found that the elution times of the analytes decreased with ACN concentration increased (Table 2). When ACN was higher than 5%, catalpol peak was overlapped with the solvent peak. However, when less than 5% of ACN was used in the mobile phase, more late AU and GPA were eluted. All the three analytes can be eluted within 10 minutes under 5% of ACN. In order to shorten the running time for compounds behind them, we adopted 5% of ACN in the final mobile phase system. The final HPLC conditions that we adopted to determine CA, AU, and GPA in our study were summarized in Table 1.

**Figure 1:**
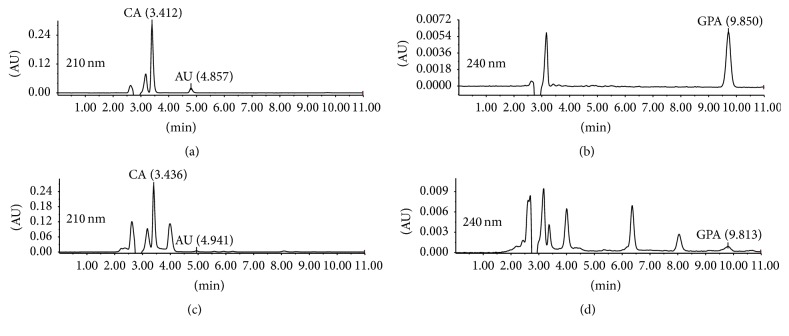
HPLC-UV chromatographs of standard mixture ((a), (b)) and* R. glutinosa* extract ((c), (d)).

Using the optimized conditions, we further tested our HPLC-UV conditions by checking its linearity, precision, stability, and recovery (Table 3).  Figure 1 showed that standards of CA, AU, and GPA were eluted separately at 3.412 min, 4.857 min, and 9.850 min. Their corresponding compounds in the sample extract were detected at 3.436 min, 4.941 min, and 9.813 min, respectively. The linear regression equations obtained were as follows: CA, *y* = 0.9707*x* + 6.7723, *R* ^2^ = 0.9997; AU, *y* = 0.9952*x* + 6.8458, *R* ^2^ = 0.9999; and GPA, *y* = 1.0136*x* + 7.5797, *R* ^2^ = 0.9996. For the precision estimation, a sample extract was assayed successively for 6 times. The precisions were evaluated as the relative standard deviation (%, RSD) and, calculated for CA, AU, and PGA, were 0.14%, 2.65%, and 2.05%, respectively. This indicated that our instrument was in good precision condition. Repeatability was obtained via parallel preparation of six sample extracts from the same batch. RSD for CA, AU, and PGA was 0. 25%, 3.08%, and 2.41%, respectively, which indicated that our extraction method was reproducible. To check the stability of extracts, the same sample extract was assayed separately at 0, 2, 4, 8, 12, 16, and 24 h after extraction. The RSD values for CA, AU, and PGA were 1.46%, 2.38%, and 0.43%, respectively, indicating that our sample extract remained stable at least till 24 hours. The extraction recovery was determined by comparing the content of the compound extracted from the samples with the content of compound from nonextracted standard solutions at equivalent concentrations. To test the sample extraction recovery, one mL of the standard solution containing 4.824 mg of CA, 0.225 mg of AU, and 0.0134 mg of GPA, respectively, was added into one gram of leaf powder at the beginning of the extraction. The recovery rates for standards CA, AU, and PGA were 102.59%, 98.29%, and 101.96%, respectively. The calculated LOD (limit of detection) at S/N > 3 was, separately, 1.3333*E* − 04 mg/mL for CA, 1.8170*E* − 04 mg/mL for AU, and 4.9926*E* − 05 mg/mL for GPA.

### 3.2. The Content of Catalpol, Aucubin, and GPA Changed with the Leaf Development

**Figure 2:**
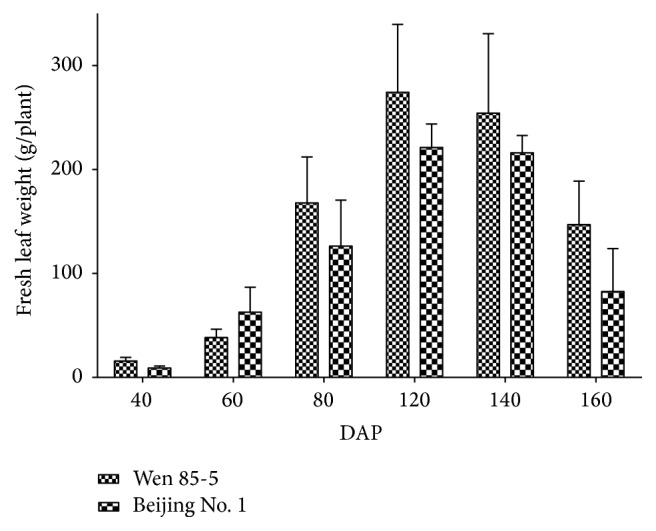
Changes of fresh weight of* R. glutinosa* leaves during the growth period.

**Figure 3:**
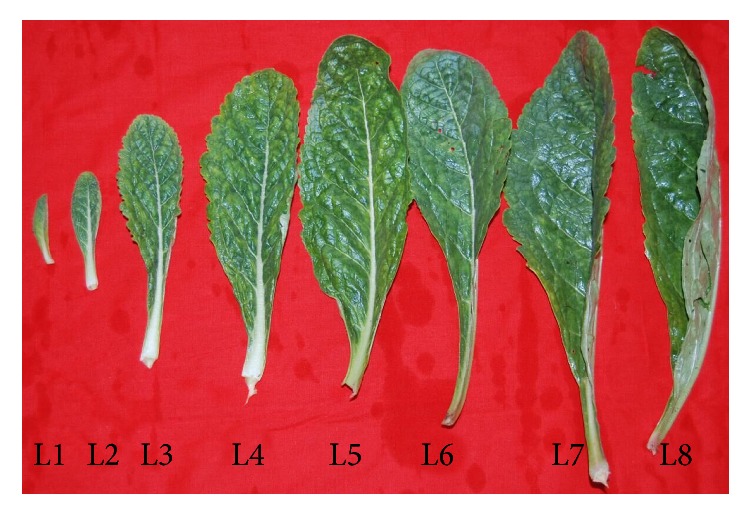
Leaf samples of* R. glutinosa* used in the study. L1–L8 represented leaves located on the top position of the stem and downwards.

**Figure 4:**
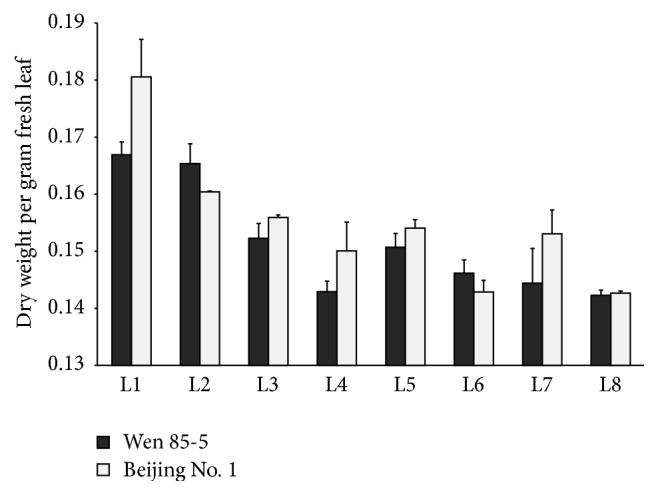
Dry weight of* R. glutinosa* L1–L8 leaves.

**Figure 5:**
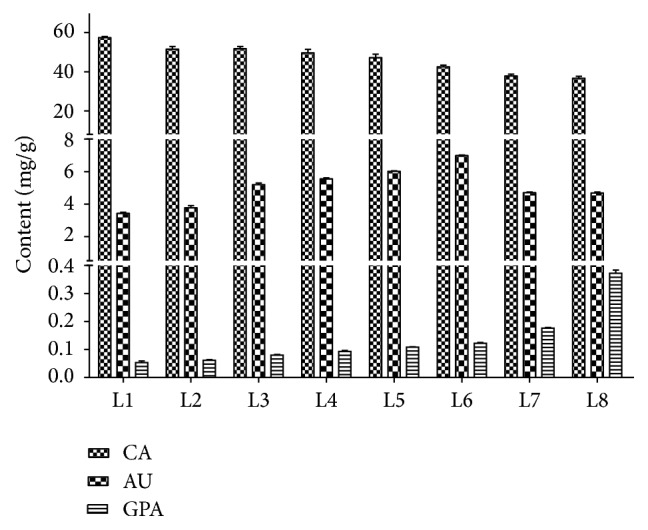
Content of catalpol, aucubin, and geniposidic acid in different developmental stages of Wen 85-5 leaves.

**Figure 6:**
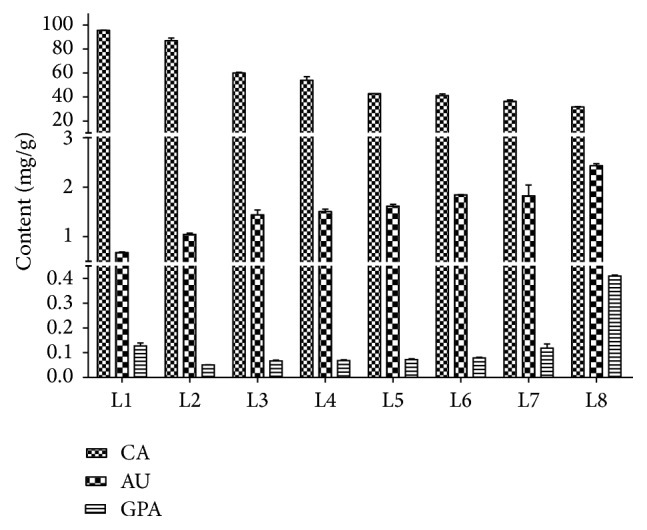
Content of catalpol, aucubin, and geniposidic acid in different developmental stages of Beijing No. 1 leaves.

*R. glutinosa* is a perennial herb with cespitose leaves on its shortened stem. The leaf biomass of* R. glutinosa* cultivars increased greatly from the seedling stage till 120 DAP (days after plantation) and then deceased after mid-September (140 DAP), partially due to ongoing senescence of bottom leaves (Figure 2). However, the top eight positions of leaves still stayed green and varied in leaf size, which can represent the different developmental stages of leaves (Figure 3). The 8th leaves, counted downwards from the uppermost leaves, looked similarly in their size and appearance status as the lower positions of unsenescent leaves. However, their dry weight (dry weight per gram fresh weight) changed dynamically with leaf development (Figure 4). Overall, leaf dry weight decreased with the leaf development and increased for L5 and then decreased in the rest of older leaves. L1–L8 had about 0.14–0.18 grams of dry biomass per gram fresh biomass. In this paper, we just focused on the contents of CA, AU, and GPA in the top eight positions of leaves and investigated their relationship with the leaf developmental stages. Although the contents of CA, AU, and GPA were somehow different between Wen 85-5 and Beijing No. 1, the changing trends of these three iridoid glycosides with the leaf developmental stage were very similar between the two cultivars, indicating no genetic difference in developmental regulation of these three iridoid glycosides (Figures 5 and 6). Generally, catalpol, a major bioactive iridoid glycoside of* R. glutinosa*, was higher in younger leaves and decreased with the leaf development, which was consistent with the result of Ji et al. [16]. Likewise in* R. glutinosa* root [18], aucubin in the leaf was still much lower than catalpol, which was found by Piątczak et al. [23]. The changing trend of aucubin among different developmental stages of* R. glutinosa* leaves was distinct from catalpol; that is, the older the leaves, the higher the aucubin in them. It was found that aucubin was one of the intermediates for catalpol biosynthesis in* Scutellaria albida *and* Paulownia tomentosa* [24]. The opposite metabolic profiling of aucubin and catalpol in* R. glutinosa* leaf indicated that catalpol might not be synthesized via aucubin; however, this needs to be further investigated. GPA was detected to be present in* R. glutinosa* leaves and the lowest analyte in all the developmental stages of* R. glutinosa* leaves. Like aucubin, GPA was increased with the leaf development and higher in older leaves.

## 4. Conclusion

In this paper, we determined contents of catalpol, aucubin, and geniposidic acid in different developmental stages of* R. glutinosa* leaves using the optimized HPLC-UV conditions and further compared their changing trend with the leaf development. Our results showed that aucubin and GPA in* R. glutinosa* leaves were much lower than catalpol and had the increasing trend with the leaf development, which was different from catalpol. This work provided an important basis for future exploitation of* R. glutinosa* leaves such as isolation of interesting metabolites, for example, the high concentration compound present at 4.16, which was further identified by HPLC-LC-MS method. It also provided a model to study the relationship of aucubin and catalpol metabolisms in* R. glutinosa*.

## Figures and Tables

**Table 1:** Optimization of HPLC-UV conditions for three analytes.

Chromatographic factor	Compared parameter	Optimized conditions	Main advantages
Column	Phenomenex Hydro_RP (4.6 mm × 250 mm, 4 *μ*m), Kinetex-C18 (4.6 mm × 100 mm, 2.6 *μ*m)	Kinetex-C18	Improved column efficiency, speed, separation, and sensitivity

*λ*_UV_	203~210 nm for CA and AU, 235~240 nm for GPA	210 nm for CA and AU; 240 nm for GPA	No difference in 203~210 nm; less miscellaneous peaks and stabler baseline at *λ* 240 nm

Mobile phase	Methanol-H_2_O, ACN-H_2_O	ACN-H_2_O	Better peak shape and separation, stabler baseline
	Adding or not adding of formic acid in H_2_O	(0.1% formic acid)	Better peak shape and separation for GPA; no obvious influence on CA and AU
	Linear gradient or isocratic	Isocratic	Smooth baseline, better separation, less miscellaneous peaks

ACN : H_2_O (0.1% FA)	1 : 99, 2 : 98, 3 : 97, 5 : 95, 7 : 93, 10 : 90	5 : 95	Shorter elution time; however, CA overlapped with solvent peak when ACN was >5%; longer elution time for AU and GPA when ACN was less than 5%

**Table 2:** Elution time of three analytes under different concentrations of ACN.

% of ACN	Rt (min) of CA	Rt (min) of AU	Rt (min) of GPA
1%	13.651	22.131	31.184
2%	7.898	11.408	23.006
3%	4.361	7.023	14.070
5%	3.436	4.941	9.943
7%	≤Rt0	3.427	8.677
10%	≤Rt0	≤Rt0	6.181

Note: Rt0 represents solvent peak, about 3.13~3.17.

**Table 3:** Validation of the optimized HPLC-UV conditions for three analytes.

Iridoid	Calibration curve	Correlation (*R* ^2^)	Precision (RSD%)	Reproducibility (RSD%)	Stability (RSD%)	Recovery (%)	LLOD (mg/mL)
Catalpol	*y* = 0.9707*x* + 6.7723	0.9997	0.14	0.25	1.46	102.59	1.3333*E* − 04
Aucubin	*y* = 0.9952*x* + 6.8458	0.9999	2.65	3.08	2.38	98.29	1.8170*E* − 04
GPA	*y* = 1.0136*x* + 7.5797	0.9996	2.05	2.41	0.43	101.96	4.9926*E* − 05
